# Design and analysis of a 60 GHz high gain wideband magneto electric dipole antenna array based on trapped printed gap waveguide technology

**DOI:** 10.1038/s41598-025-08589-9

**Published:** 2025-07-02

**Authors:** Haitham Hamada, Mohamed Mamdouh M. Ali, Shoukry I. Shams, Ashraf A. M. Khalaf, A. M. M. A. Allam

**Affiliations:** 1 Electrical Engineering Department, Higher Technological Institute, HTI, 10th of Ramadan city, Egypt; 2 Department of Electrical Engineering, Faculty of Engineering, Assiut University, Assiut, Egypt; 3 Department of Electrical and Computer Engineering, Concordia University, Montreal, QC Canada; 4 Electrical Engineering, Minia University, Minia, 61519 Egypt; 5 Information and Engineering, Technology Department, German University in Cairo, Cairo, Egypt

**Keywords:** Engineering, Electrical and electronic engineering

## Abstract

This paper introduces an innovative design and analysis of a magneto-electric dipole antenna exhibiting high-gain, ultra-wideband operation, and stable radiation characteristics in the 60-GHz mm-wave band. Furthermore, the trapped printed gap waveguide (TPGW) technology is presented as a low-cost, minimal-loss, and low-dispersion guiding structure to feed the proposed antenna. The antenna covers a relative matching bandwidth of over 33.33% from 50 to 70 GHz with a maximum gain up to 8 dBi. In addition, the antenna is integrated with a perforated dielectric substrate layer lens on the antenna’s broadside location, enhancing the gain by an average of 3 dB along its entire operational bandwidth. Moreover, an efficient approach for designing a large ME dipole antenna array and its corporate feeding network is presented. Both ME-dipole sub-arrays and the out-of-phase power divider with WR-15 standard interface are designed and studied separately, where a systematic design procedure is presented to obtain initial design parameters. A 2 × 2 planar antenna array is designed and implemented, featuring proper integration between the radiating elements and a differentially fed wide-bandwidth TPGW power divider. Then, the operation of the individual components has been assessed using simulation and measurements. Furthermore, an in-depth mathematical analysis is presented to investigate the potential resonance conditions arising from disparities in complementary components. Consequently, a proposed solution is provided to break the resonance loop and shield the two opposing sub-arrays. The 2 × 2 array of ME-dipoles has overall dimensions of 1.6$\lambda _0$ $\times$1.4$\lambda _0$ and demonstrates an impedance bandwidth ($|S_{11}| <$– 10 dB) exceeding 33.33$\%$ at 60 GHz, with a peak gain of over 18 dBi.

## Introduction

The demand for wireless communication and its customized services has witnessed significant growth over the past few decades. The 5G wireless communication standard leverages the millimeter-wave spectrum, which offers wide bandwidth for high-data-rate applications^1,2^. The mm-wave frequencies are well-suited to meet the requirements for wide operating bandwidths, making them ideal for 5G mobile communications and future 6G standards.

Millimeter-wave transmission in the 60 GHz range is a promising approach to meet the demanding requirements of future wireless systems^3,4^. The 60 GHz band is recognized as an ideal candidate for short-distance, high-data-rate applications, including high-definition streaming^1,2^, virtual reality, and the Internet of Things (IoT)^5^. The deployment of the 60 GHz band represents a significant advancement toward enabling more secure wireless communications. The newly unlicensed 5G band offers a wide bandwidth of 14 GHz, spanning from 57 to 71 GHz, although it presents several associated challenges^6^. This band accommodates a diverse array of applications, prompting significant efforts to develop mm-wave components and antennas that operate effectively within this frequency range.

Many antennas were proposed as a composition of two complementary sources using a slot with two wires controlling E-and H-plane radiation patterns^7,8^. Moreover, a magneto-electric (ME) dipole is considered a complementary antenna in which an electric dipole and a magnetic dipole are located orthogonally and excited simultaneously. In addition, the antenna demonstrates wide bandwidth, enabling various applications such as mobile and millimeter-wave communications^9^. Different ME dipole configurations with various feeding techniques are designed to provide low-profile antennas with wide bandwidth^10–12^. On the other hand, radio waves in the mm-wave band have high atmospheric attenuation at 60 GHz, which reinforces the need for high-gain antennas^13^.

Various studies have proposed different approaches for designing wide-bandwidth antennas with high gain in millimeter-wave frequency bands. Antennas based on low-temperature co-fired ceramic (LTCC) technology are commonly used to cover the 60 GHz band with high gain; however, these designs are often associated with high fabrication costs and complex assembly processes^14,15^. Another method involves loading an antenna with a Frequency Selective Surface (FSS) superstructure, but this approach suffers from a limited bandwidth over the operating frequency range^16^. Substrate Integrated Waveguide (SIW) feeding systems have been employed to provide antennas with wide bandwidth, stable radiation patterns, and low cross-polarization levels^17,18^. However, the SIW structure supports TE modes inside a dielectric medium, leading to dispersion and dielectric losses. In contrast, Gap Waveguide (GW) technology offers a low-loss solution for mm-wave bands compared to SIW and microstrip lines, with the added advantage of requiring no electrical contacts.

The printed gap waveguide (PGW) represents a lightweight and cost-effective technology that has recently been employed to enhance mm-wave antenna performance^19,20^. Additionally, PGW incorporates a shielded feeding mechanism, which minimizes radiation losses and prevents interference from the feeding structure on the radiation characteristics of the main radiating element. Various approaches utilizing PGW technology have been developed to improve antenna gain and bandwidth. However, dielectric resonator antennas (DRA) with wide bandwidths are often associated with significant fabrication complexity^21,22^. A proposed Fabry-Perot cavity design featuring two FSS layers based on PGW is introduced, which can operate with or without a reflector to control gain^23,24^. Despite substantial efforts to create high-gain antennas in the mm-wave band using various technologies, there remains considerable room for improvement. Many existing designs in the mm-wave band either experience significant losses or are plagued by notable fabrication complexities. Furthermore, trapped GWs can offer an effective remedy for handling challenging manufacturing operations in high-frequency circuits^25,26^.

Within the scope of this work, we propose the design and analysis of a broadband magneto-electric dipole antenna based on TPGW technology to achieve a stable wide bandwidth and high gain suitable for 60 GHz mm-wave applications. This antenna represents a significant advancement over the challenges of high manufacturing costs and complex designs. The TPGW structure feeds the ME dipole antenna through a bow-tie-shaped slot, resulting in a printed low-profile configuration. Additionally, a perforated dielectric substrate lens has been integrated on top of the ME antenna to enhance gain. A 2 × 2 array has been designed to provide increased gain by placing the lens above the elements of a 4-element array. Utilizing TPGW technology allows for a reduction in the spacing between the feeding network lines and between the directly fed radiating elements, enabling distances that are less than the wavelength. Through extensive investigation, we identified that the primary cause of the network’s inability to achieve the desired bandwidth is significant coupling between the antenna sub-arrays within the overall structure. Consequently, we will present a comprehensive mathematical analysis to study the impact of practical asymmetry between the individual components within the proposed design. The final step will involve presenting an effective solution to mitigate the discrepancies among the practical components to improve the overall assembly response.

This article is organized as follows: section “The antenna geometry and design” presents the TPGW structure, beginning with the first subsection that describes the feeding mechanism. The second subsection details the antenna configuration and the proposed radiator geometry. Section “ME dipole antenna array” introduces the ME dipole antenna array. A mathematical analysis justifying the results and explaining the excitation of cavity mode resonances is provided in section “Mathematical analysis of cavity mode resonances excitation”. Section “Modified array structure: mutual coupling solution” discusses the solution to the asymmetry problem of the ME dipole antenna array, followed by evaluation and comparison in section “Evaluation and comparison”. Finally, section “Conclusion” summarizes and concludes the findings of this article.

## The antenna geometry and design

In this section, we present the geometry of the proposed magneto-electric dipole antenna alongside the TPGW feeding line. The unit cell of the TPGW structure will be analyzed to ensure effective operation within the target frequency band. This analysis will be illustrated for both the unit cell and an array of unit cells in the subsequent subsection. Additionally, a separate subsection will provide a detailed description of the antenna configuration, thereby completing the discussion on the antenna geometry and design.

### The ridge gap waveguide unit cell design

**Fig. 1:**
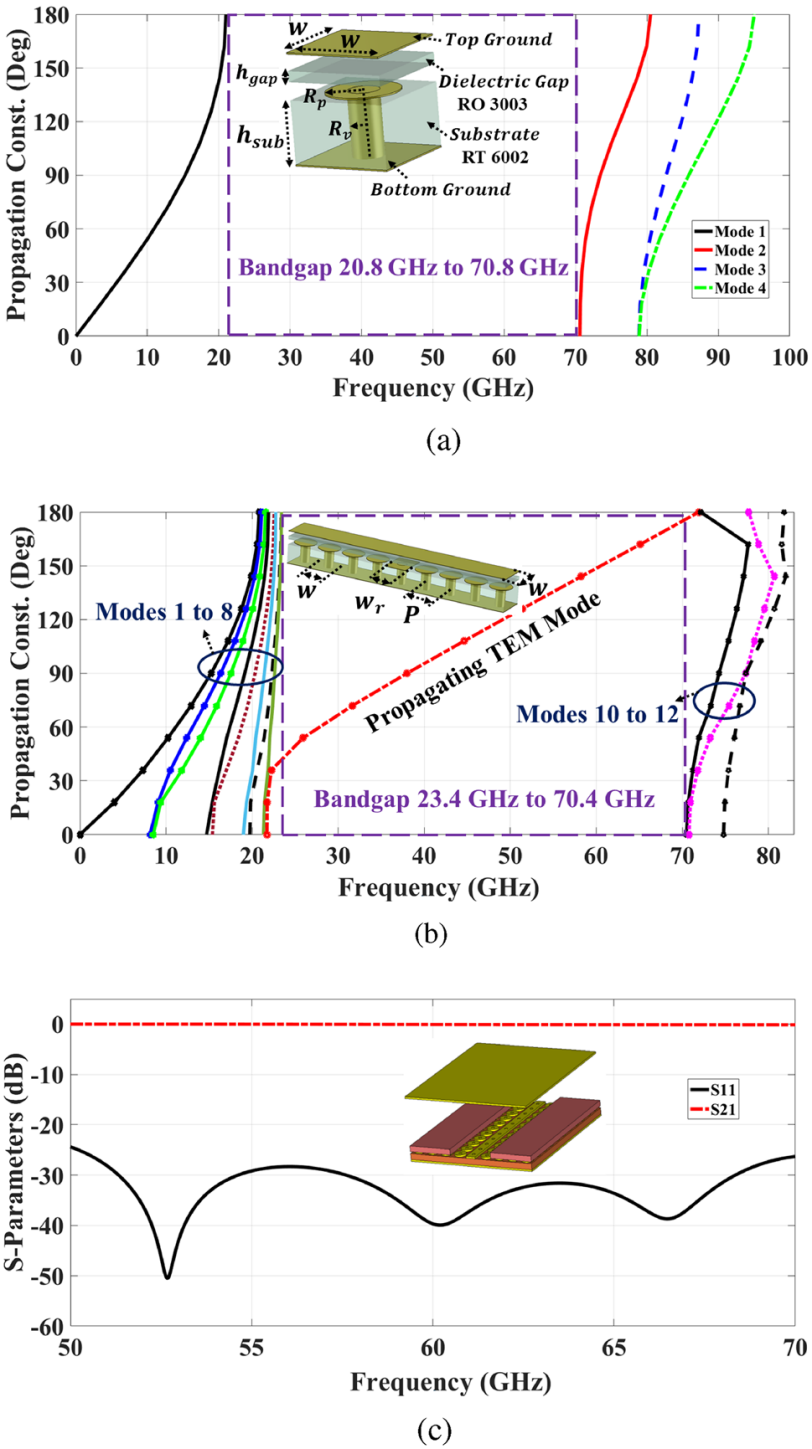
Dispersion diagram of (**a**) Unit cell. (**b**) Row of unit cells. (**c**) Simulated transmission parameters for a basic TPGW structure.

The key characteristic of the gap waveguide (GW) concept lies in the construction of a periodic structure surrounding the ridge to prevent leakage. A wide range of unit cell shapes can be developed to achieve different operating frequency ranges. Moreover, the bandwidth depends on the type and shape of the applied unit cell. TPGW technology utilizes bandgap periodic structures, such as mushroom cells, to effectively package the feeding lines and inhibit propagating modes within the bandgap of these structures. Furthermore, a structure with a mushrooms can form a high impedance surface, also known as an AMC surface, which prevents wave propagation within the surface. However, the ridge allows wave propagation in specific directions. This mushroom version is more compact and appropriate for mm-Wave applications and other packaging techniques. Figure 1a illustrates the basic analysis configuration of the mushroom unit cell, which has a height of $h_{sub}$ and features two copper-annealed plates on the top and bottom. A substrate layer with a relative permittivity of 2.94 (Rogers RT6002) and a thickness of 0.762 mm is positioned directly above the bottom ground plane to support the metal-plated vias. The shape of mushroom patches may differ. It’s a printed representation of a ridge gap waveguide that has reduced conduction losses. In this configuration, a circular patch on top of the pin acts as an artificial magnetic conductor (AMC) that can be designed to provide the desired bandgap. A further advantage of using this unit cell is its wide bandwidth. Furthermore, this makes the fabrication process easier and simpler than the other gap waveguides. A dielectric-filled gap with a relative permittivity of 3 (Rogers RO3003) and a thickness of 0.13 mm separates the mushroom-like surface from the top plate.

The design of the unit cell has been extensively investigated in numerous studies, with reported cell parameters tailored to cover various operating bands^27–29^. These parameters have been selected in accordance with established guidelines from the literature, and all dimensions are summarized in Table 1. The dispersion diagram of the proposed TPGW unit cell has been simulated using the CST Microwave Studio Eigen Mode Solver with periodic boundary conditions, illustrating the bandgap characteristics of the unit cell in Fig. 1a. It is apparent that the employing of this unit cell has a wide bandwidth. The analysis reveals a broad bandgap that spans a frequency range of 20.8 to 70.8 GHz, effectively accommodating the required operating band within the millimeter-wave frequency spectrum while providing sufficient margins.

The unit cell is arranged in a linear pattern, creating a row of unit cells that surround the guiding ridge. This ridge is positioned at the center of the substrate to mitigate wave propagation in other directions, aided by the presence of the artificial magnetic conductor (AMC). Additionally, the ridge is equipped with pins that connect to the bottom ground layer to further suppress radiation beneath the structure. The arrangement maintains a periodicity *P* consistent with that of the mushroom unit cells. The operating bandwidth of the row of unit cells can be derived from the dispersion diagram shown in Fig. 1b. However, it is observed that the introduction of the ridge influences the realized bandwidth of the unit cell.

**Table 1:** Dimensions of the TPGW unit cell in mm.

Parameter	Value	Parameter	Value
$h_{sub}$	0.762	$R_{p}$	0.44
$h_{gap}$	0.13	$R_{v}$	0.19
*W*	1.22	*P*	1.06

As the ridge line supports a quasi-TEM mode, the stripline model is an appropriate method for the ridge gap waveguide to approximate the calculation of the characteristic impedance based on the ridge width and the gap height^30–32^. Additionally, increasing the line width and gap results in reduced conductive losses. This allows the line width to be maximized without exciting higher-order strip modes while maintaining a broad stopband with sufficient margins relative to the operational band^33^. However, it is crucial to prevent degradation of the characteristic impedance as the ridge width increases. Alternatively, the diameter of the vias beneath the line determines the maximum allowable width to avoid manufacturing issues. Accordingly, an additional 0.254 mm was added to each side of the pins, resulting in an overall line width of 0.889 mm. Moreover, to mitigate potential fabrication limitations associated with mushroom cell distribution, the spacing is adjusted to 0.165 mm. The detailed dimensions of the ridge line width and the spacing of the mushroom cells are provided in Table 2. Various studies utilize a specific number of unit cells surrounding a ridge to effectively prevent surface wave leakage and eliminate grating lobes in array systems^34^. The results depicted in Fig. 1c illustrate the S-parameters of the proposed TPGW structure based on the two-port analysis. Notably, the matching level consistently below -20 dB across the entire operating bandwidth of 50-70 GHz.

### Magneto-electric dipole antenna design guideline

**Fig. 2:**
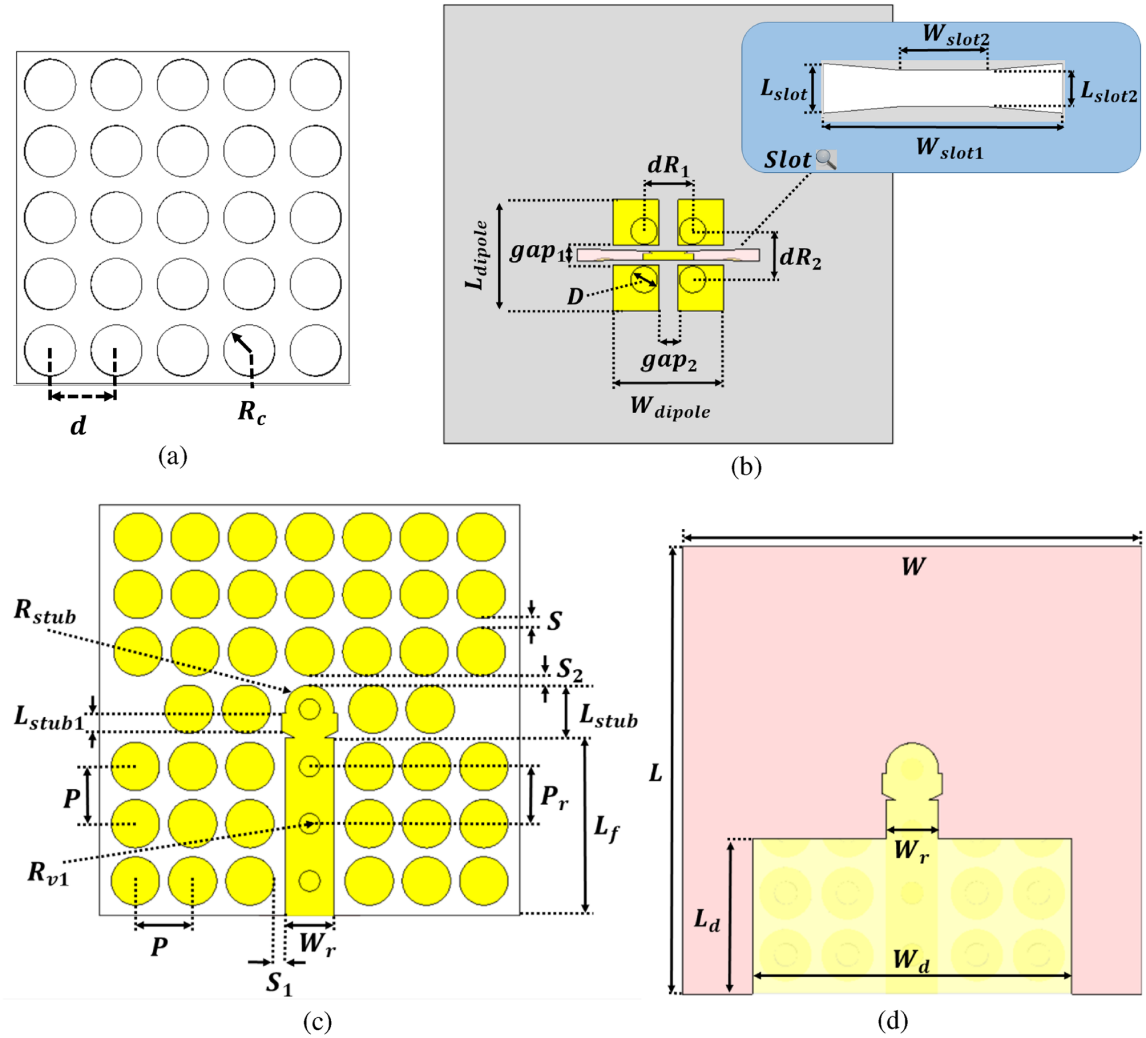
(**a**) Perforated Dielectric superstrate lens front view. (**b**) The radiating element (ME dipole). (**c**) The Bottom layer of TPGW structure. (**d**) TPGW line.

The detailed configuration of the presented antenna for mm-wave applications is shown in Fig. 2, where a magneto-electric (ME) dipole antenna is selected as the radiating element. ME dipole antennas outperform traditional radiators such as electric dipoles and slot antennas^35–37^, owing to their ability to produce a symmetrically unidirectional radiation pattern with low back radiation. In such designs, the magnetic dipole is typically realized by employing an open-ended shorted-patch antenna with vertical walls and a metallic ground plane, while the electric dipole is formed using planar horizontal patches^38,39^. In previous ME dipole designs, the electric dipole consists of a pair of patch antennas printed on the top surface of the substrate, with vertical walls substituted by four metallic vias^40^. However, achieving a proper impedance match with this configuration can be challenging when the structure is driven by a coupling aperture etched onto the ground plane. In alternative designs, planar dipoles are excited by two vias, replacing the inverted-L wires found in^7^, allowing the antenna to operate effectively at millimeter-wave frequencies^54^. Additionally, the radiating element features two dipoles, unlike the single dipole employed in^9^.

**Table 2:** Dimensions of the proposed single element antenna structure in mm.

Parameter	W	L	$w_{r}$	Gap	$gap_{2}$
Value	7.8	7.6	0.889	0.3	0.3
Parameter	$dR_{2}$	$L_{dipole}$	$W_{dipole}$	$W_{slot}$	$W_{slot2}$
Value	0.84	1.9	1.93	3.17	1.1
Parameter	D	$dR_{1}$	$L_{slot}$	$L_{slot2}$	$L_{d}$
Value	0.45	0.84	0.2	0.157	2.65
Parameter	$W_{d}$	$L_{stub1}$	$S_{1}$	S	$S_{2}$
Value	5.46	0.3	0.2	0.165	0.168
Parameter	$P_{r}$	$L_{f}$	$L_{stub}$	$R_{v1}$	$R_{stub}$
Value	1.06	3.3	0.97	0.2	0.44

In our design, the planar electric dipole consists of two pairs of horizontal patches, while the aperture between these patches serves as the alternative magnetic source, as depicted in Fig. 2b. These planar strips are printed on a Rogers RT6002 substrate with a thickness of 0.762 mm and are connected to the ground via metallic conductive vias. The radiation structure is constructed using Substrate 1, which includes four metallic pins and four horizontal patches. The ME dipole antenna is excited through a transversely narrow Bow-tie slot etched into the metallic ground layer, which is part of the TPGW feed line. This slot is shifted slightly off-center to provide both wide impedance matching and enhanced isolation bandwidth. Additionally, the TPGW feed line is terminated with a matching stub, as shown in Fig. 2c, which improves the impedance matching over the required bandwidth and ensures better overall performance of the antenna system.

The top layer of the dielectric-filled gap in the TPGW structure, which implements the trapped PGW concept, is depicted in Fig. 2d. The detailed antenna dimensions are listed in Table 2. The TPGW design employs partially filled air gaps within a conventional PGW structure. This innovative approach provides a practical solution for mitigating fabrication challenges at mm-wave frequencies, particularly in circuit implementation. This configuration is advantageous as it minimizes power consumption, making TPGW a strong candidate for mm-wave applications. The transmission line’s performance remains unaffected, as electromagnetic (EM) fields are concentrated around the line, preventing significant losses. The reflection at the air-dielectric interface ensures that EM waves are confined within the air-filled gap, enhancing isolation and reducing propagation loss. Additionally, due to the dielectric loading of mushroom unit cells, the stop-band shifts to lower frequencies without interfering with the intended operating band. The quasi-TEM mode spectrum remains stable, as the electric fields do not strongly affect the outer mushroom cells, minimizing disruptions. Another key benefit of this method is that the line’s characteristic impedance remains unchanged, simplifying the design process and maintaining consistent performance across the operating band.

**Fig. 3:**
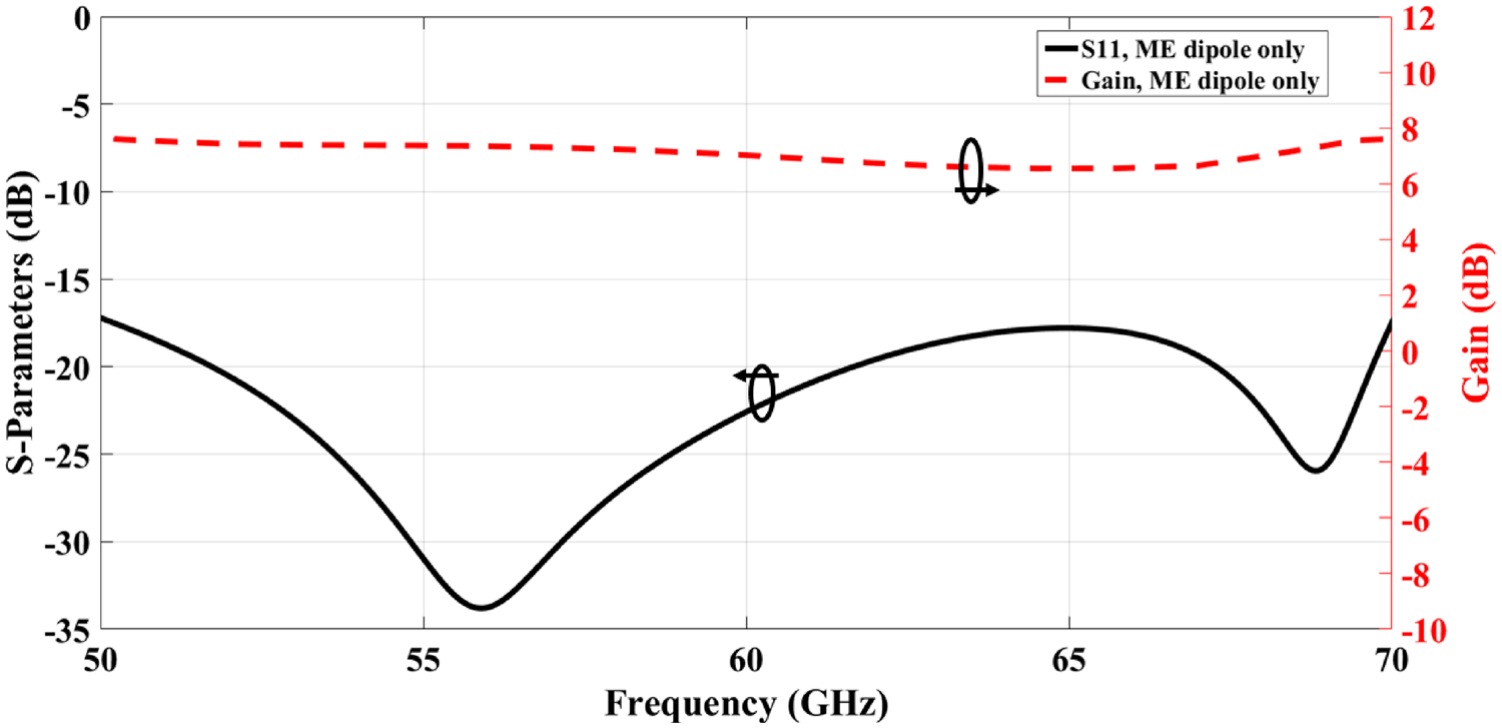
The Reflection coefficient and gain of the single element antenna structure without lens.

**Table 3:** Perforated dielectric substrate lens layer dimensions (In MM).

Parameter	Value	Parameter	Value
$h_{s}$	1.28	$R_{c}$	0.34
$h_{g}$	3.75	$d_{s}$	0.88

**Fig. 4:**
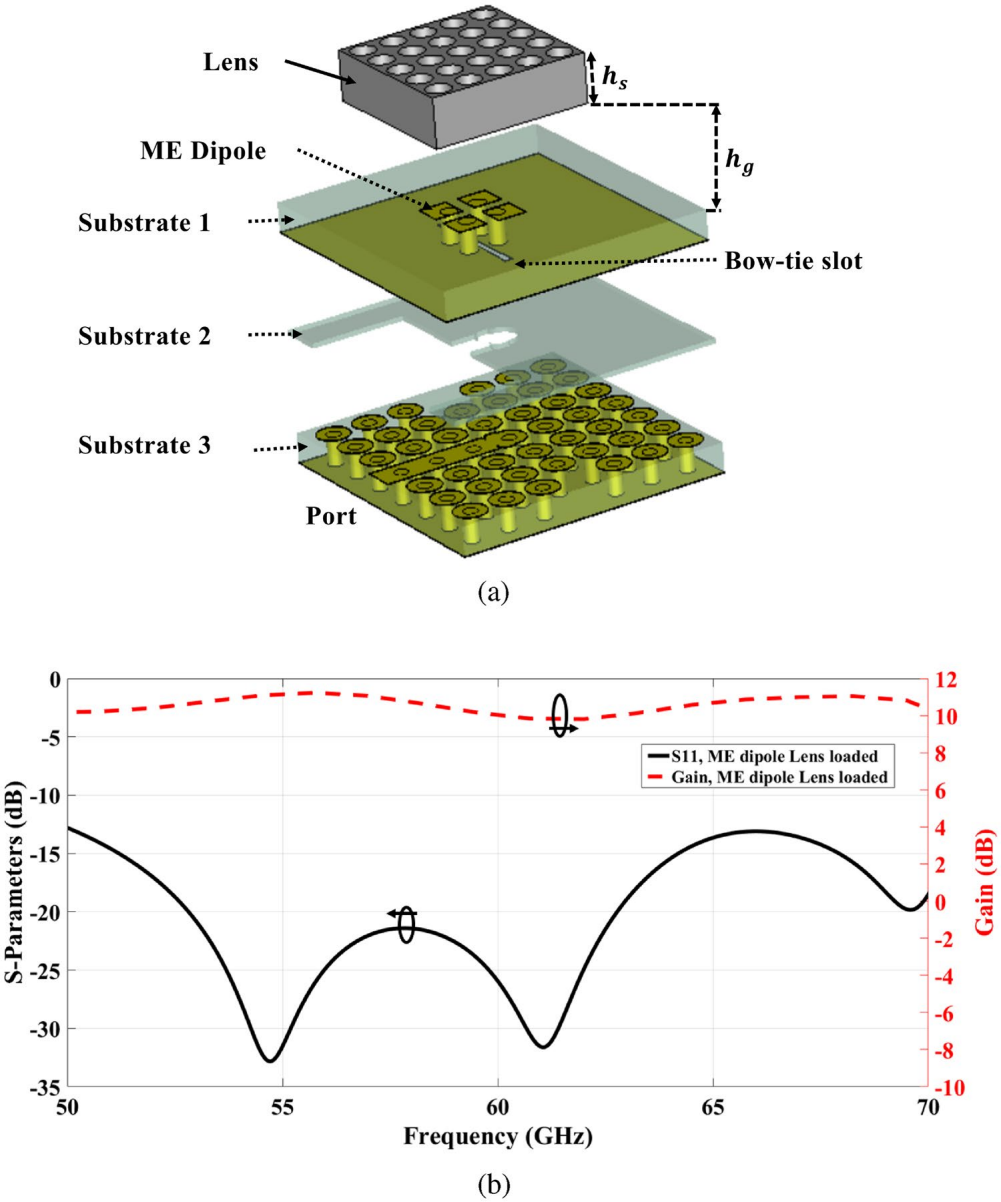
(**a**) Single element antenna structure. (**b**) The Reflection coefficient and gain of the single element antenna structure with lens.

**Fig. 5:**
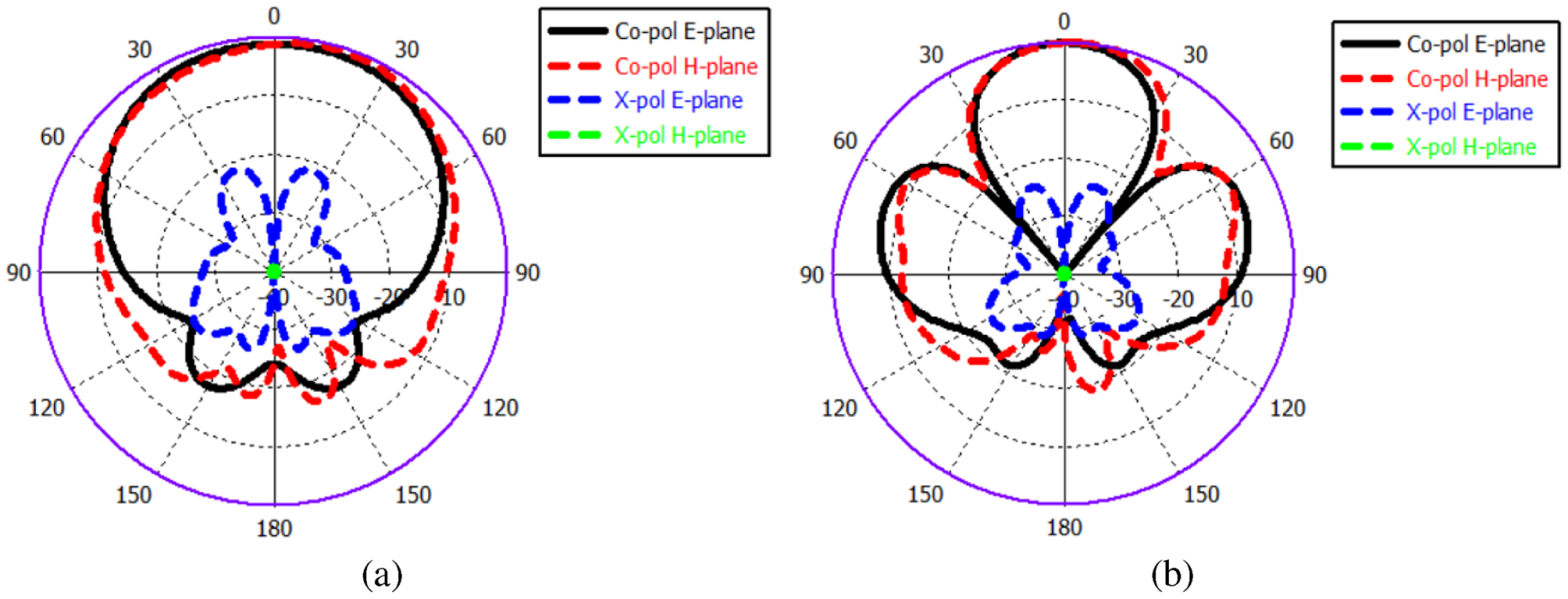
Radiation patterns of single ME-dipole antenna at 60 GHz. (**a**) Without lens. (**b**) With lens.

Additionally, a perforated dielectric Roger RO3010 substrate with a relative permittivity of 10.2 and a standard thickness of 1.28 mm is used to enhance the antenna’s gain, which acts as a lens as shown in Fig. 2a. The concept of a high-gain ME dipole antenna using such a lens was previously discussed in^41^. The lens is constructed with a 5 × 5 grid of air holes and is positioned at a distance $h_g,$ equivalent to three-quarters of a wavelength, from the ME dipole. The dimensions and distribution of the holes have a substantial impact on the antenna’s performance. Table 3 shows the optimal dimensions of the air holes of the lens layer for enhancing the gain of the ME antenna. For a single-element antenna, the simulated reflection coefficient and gain with and without lens are illustrated and compared in Figs. 3 and 4. In both cases, the single-element radiation patterns of the ME-dipole are considered as presented in Fig. 5. Evidently, the lens has no detrimental impact on the reflection coefficient, resulting in an acceptable matching level over the operating bandwidth. It is evident that the lens does not negatively impact the reflection coefficient, maintaining an acceptable matching level across the operating bandwidth. However, the lens significantly enhances the gain of the ME antenna, increasing it by up to 3 dBi. As a result, the proposed high-gain ME antenna achieves an average gain of 10–11.3 dBi over a wide frequency range. The design of a ME dipole antenna array based on this configuration will be discussed in the subsequent sections.

## ME dipole antenna array

### Two elements ME dipole antenna array

**Fig. 6:**
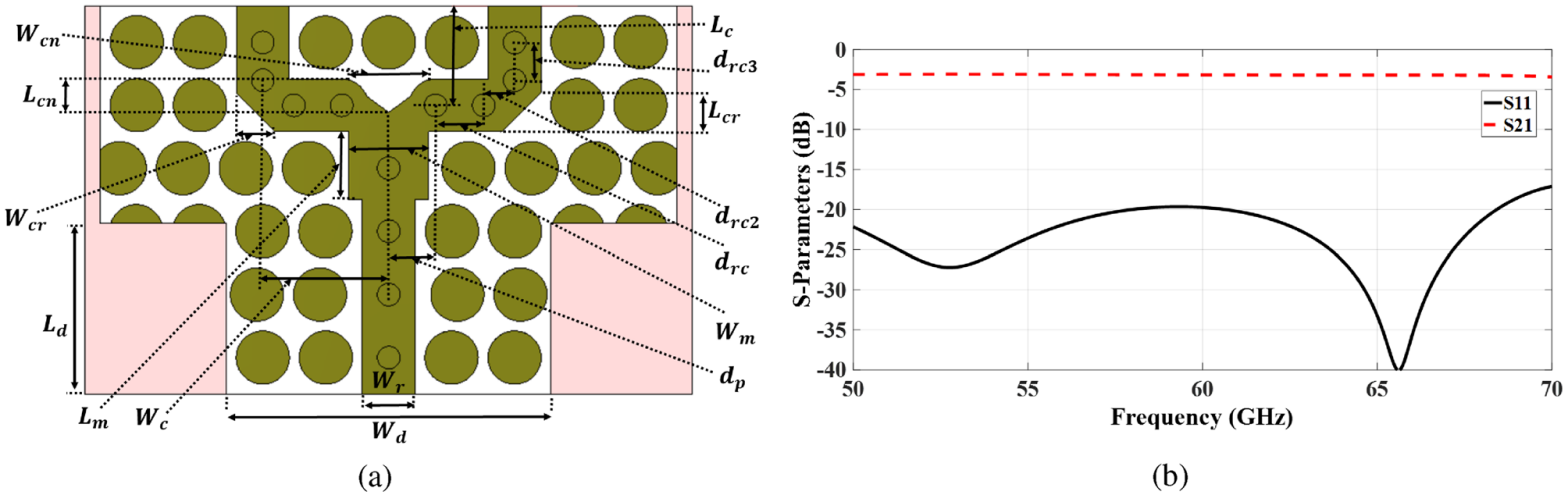
The 1-to-2 power divider (**a**) Configuration. (**b**) Reflection coefficient and transmission coefficient.

This section presents the design of a 2-element ME dipole antenna sub-array using TPGW technology. A 1 $\times$ 2 feeding network is initially designed to provide an appropriate matching level of $<-20$ dB over 50–70 GHz, where Fig. 6a demonstrates the schematic view along with all the needed design parameters listed in Table 4. In this design, a TPGW-based power divider structure is implemented using the same unit cell configuration. A 1 $\times$ 2 power divider is constructed with a two-way feeding network. The 1 $\times$ 2 feeding network is based on a T-shaped power divider and quarter-wavelength impedance matching level transformers. Parallel connections have input impedances that are approximately half of the characteristic impedances of single lines. The strip line equation provides a decent approximation for the initial value of the characteristic impedance^27,42^. Starting dimensions of the calculated matching transformers based on Chebyshev polynomials can be obtained. Additionally, a triangular slice has been cut at the junction of the two branches to improve the matching level.

Since the 2-element ME dipole antenna sub-array will be excited with equal amplitude and phase by the 1 $\times$ 2 feeding network, the element spacing is selected to be about half the wavelength at 60 GHz to prevent the grating lobe problem. 1 $\times$ 2 power divider design preserves and takes into consideration the distribution and arrangement of unit cells surrounding the ridges in order to maintain the separation between them and the ridges. The fine-tuning separation is assessed, and at least one unit cell row is maintained between these neighbouring cavities under the slots to prevent mutual coupling. As depicted in Fig. 6b, the corresponding S-parameters of the 1 $\times$ 2 power divider demonstrate adequate impedance matching within the desired frequency range. The simulated results show a uniform power distribution. Additionally, the transmission coefficient for each port is observed to be approximately − 3.2 dB across the 50–70 GHz range, which indicates that the insertion loss is about 0.2 dB.

**Table 4:** Dimensions of the 1 to 2 power divider configuration in mm.

Parameter	Value	Parameter	Value
$W_{m}$	1.34	$W_{cn}$	1.52
$L_{m}$	1.14	$L_{cn}$	0.56
$W_{cr}$	0.65	$W_{c}$	2.1
$L_{cr}$	0.65	$L_{c}$	1.67
$d_{p}$	0.78	$d_{rc2}$	0.52
$d_{rc}$	0.8	$d_{rc3}$	0.63

**Fig. 7:**
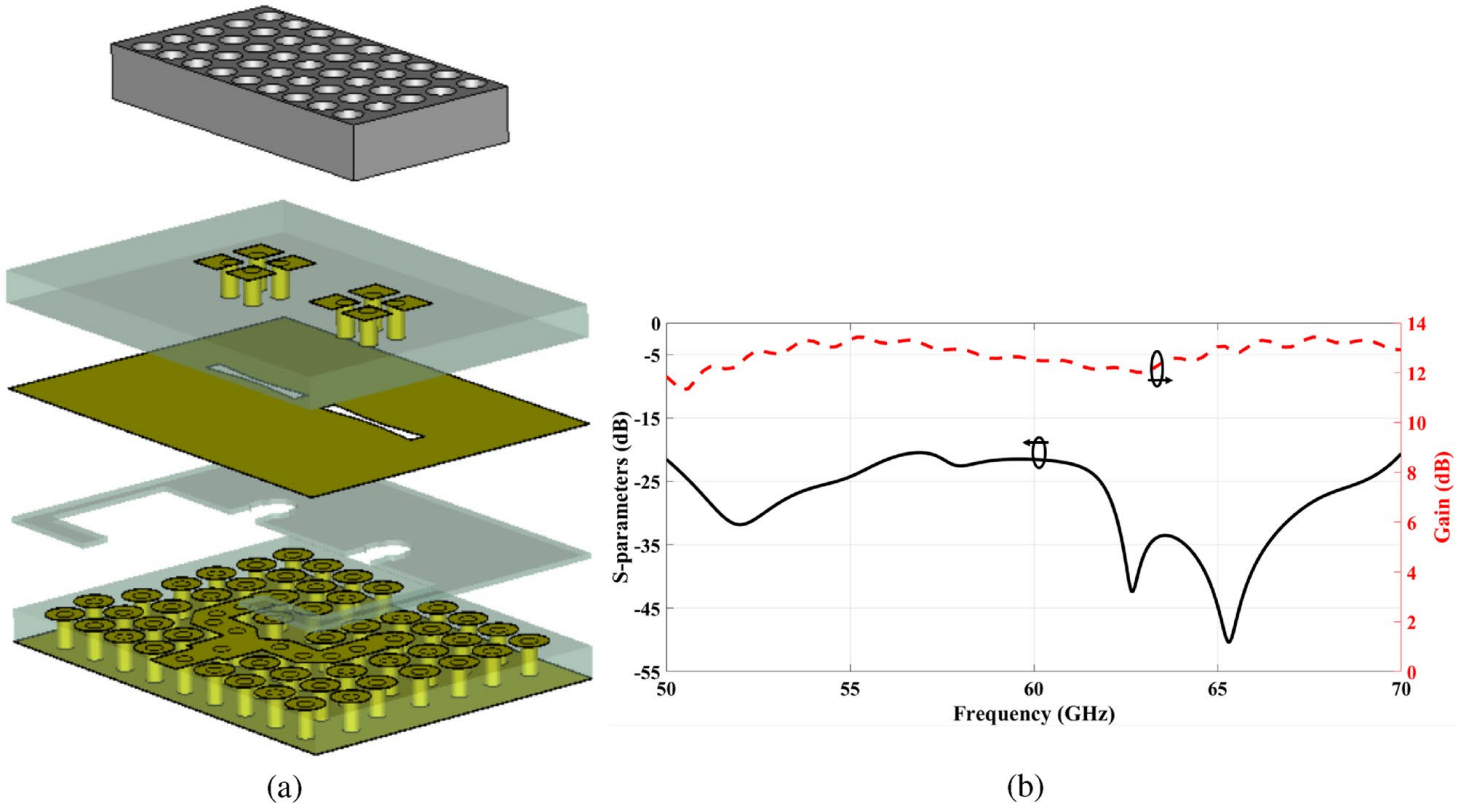
The proposed antenna. (**a**) Two elements antenna structure. (**b**) Reflection coefficient and gain.

**Fig. 8:**
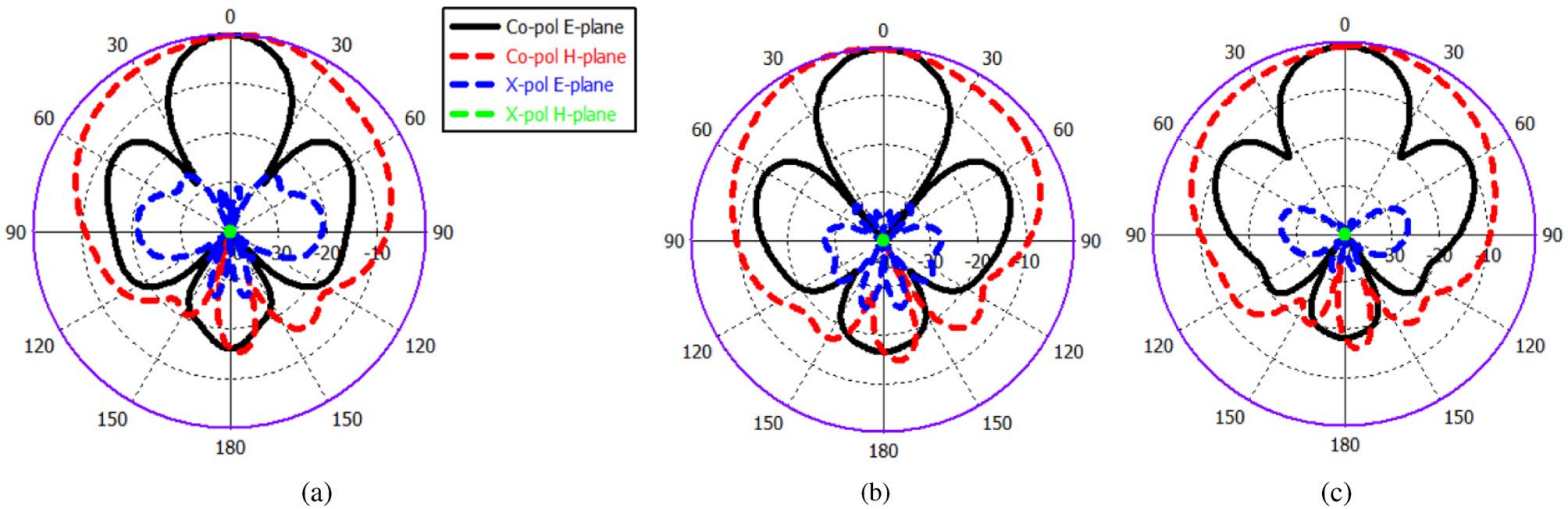
Radiation patterns of two ME-dipole element antenna (**a**) at 60 GHz. (**b**) at 55 GHz. (**c**) at 65 GHz.

The aforementioned power divider is utilized to feed two ME dipole antenna elements, as illustrated in Fig. 7a. The reflection coefficient of the presented array is shown in Fig. 7b. It is evident that the design effectively preserves the operating bandwidth of < − 20 dB. To mitigate grating lobes, the spacing between the ME dipole antenna elements is maintained at approximately 0.85 $\lambda _0,$ where $\lambda _0$ represents the free-space wavelength at 60 GHz. The two-element array results in a narrower beam in the E-plane, while a wider beam is produced in the H-plane, as demonstrated by the element pattern in Fig. 8.

### 2 $\times$ 2 ME dipole antenna array

Most feeding networks are designed with a fixed input impedance termination, which can either be a frequency-independent load that matches the characteristic impedance of the guiding lines or an isolated element whose input impedance is tuned to the center frequency of the operating band. Such an array design often overlooks discontinuities in the feeding network and the effects of mutual coupling on the input impedance of the elements, potentially leading to undesirable results. This feeding network serves as a preliminary step for full optimization, aiming to develop a new design that addresses these issues. However, this process necessitates a wide range of optimization parameters, which significantly slows down the optimization process. Additionally, the presence of radiating elements in the simulation considerably increases computational complexity. The substantial numerical size of the simulations and the requirement for significant storage space further complicate the problem. Moreover, the incorporation of the TPGW’s periodic structure adds to the overall computational challenges.

The structure is designed in stages, beginning with the analysis of a two-ME dipole sub-array. Each two-element sub-array structure is optimized iteratively, adjusting various levels and dimensions. Through separate optimization processes, each of these two-element feeding networks is designed to achieve the appropriate impedances at the ports of their respective sub-array feeding networks. Consequently, the two opposite sides of the feeding network are positioned 180 degrees apart, resulting in symmetry around the x-axis. It is important to note that this framework limits cross-polarization in the array. Furthermore, the 2 $\times$ 2 sub-array consists of two paired two-element sub-arrays, with the lengths of the feeding network arms being twice as long as in the previous design. This method effectively divides the complexity into multiple optimization tasks for each two-element feeding network.

**Fig. 9:**
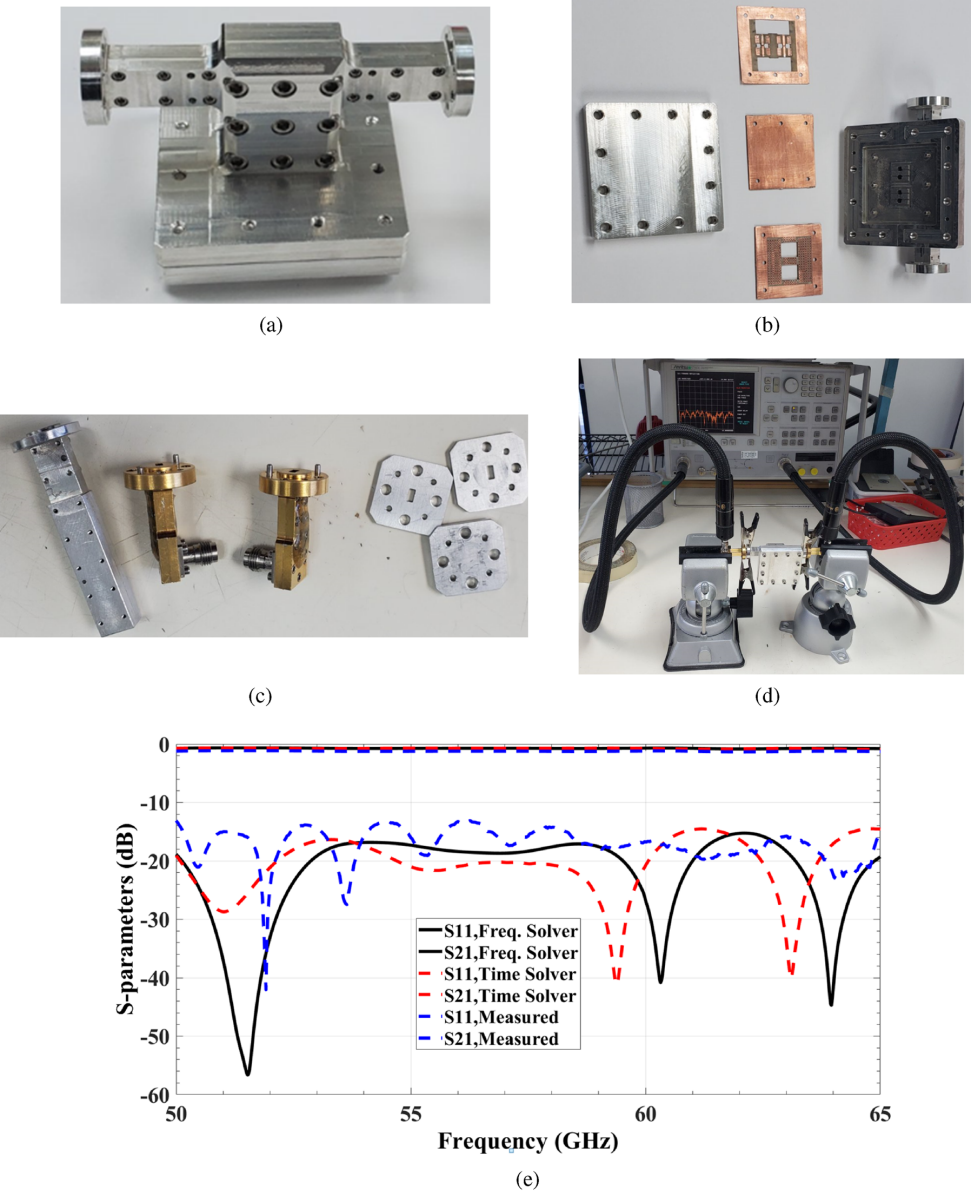
Out-of-phase power divider with WR-15 interface. (**a**) The DPD whole structure prototype. (**b**) The DPD whole structure prototype layers. (**c**) Photograph of the individual components of the DPD measurement setup process. (**d**) Photograph of the DPD measurement setup process. (**e**) Simulation and measurement results.

**Fig. 10:**
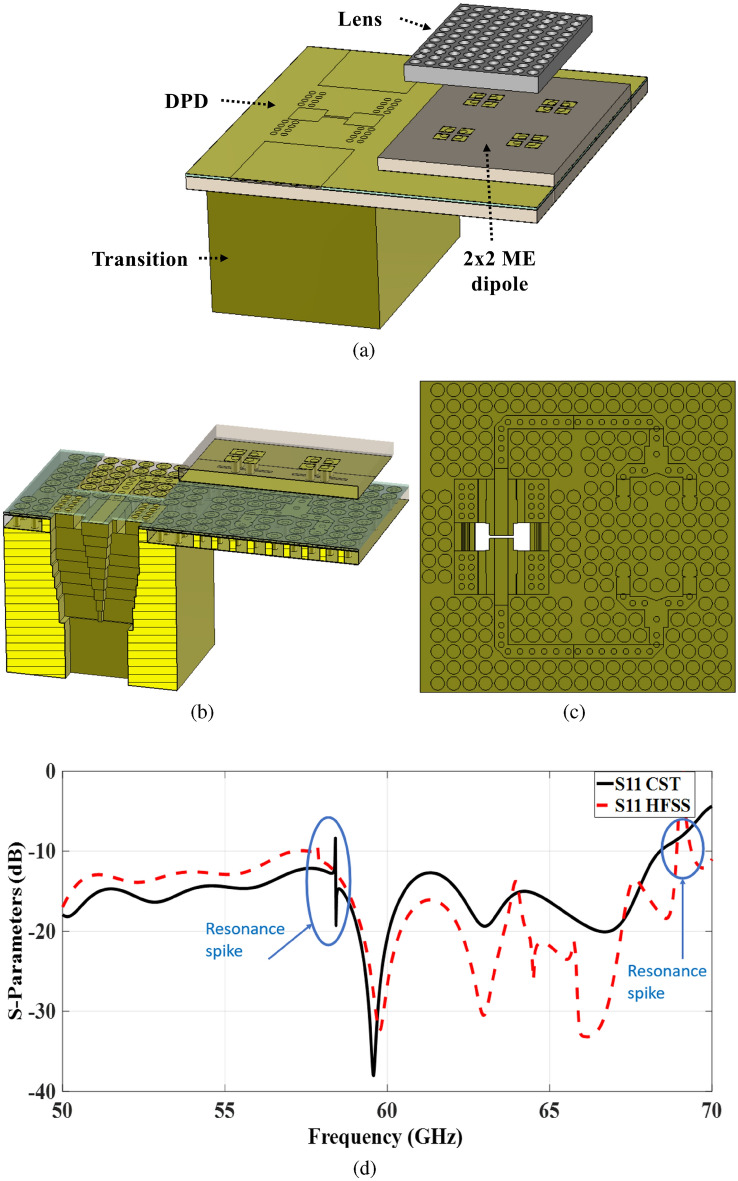
2 $\times$ 2 ME dipole antenna array structure. (**a**) 3-D view of the whole structure. (**b**) 3-D view with cutting plane. (**c**) Top view of the TPGW feeding network. (**d**) Simulation results.

The symmetry inherent in the design also has the potential to reduce overall processing time and minimize the number of optimization trials required for each two-element configuration. Moreover, the entire array antenna is excited via a standard V-band rectangular waveguide (WR-15) at the bottom of the structure. The concept and design details of the differential power divider with the standard WR-15 interface are given in^25^. An out-of-phase power divider with a standard WR-15 interface is designed in the desired operating bandwidth and implemented to validate its performance, as depicted in Fig. 9. The power divider is based on a TPGW that uses aperture coupling to maintain a stable 180-degree phase imbalance. The power divider and transition have been implemented and tuned to reduce the reflection coefficient at the input WR-15 port within the specified frequency range. To design the transition between two waveguides with different cross-sections, multi-section transformers have been employed. These matching transformers have been designed to serve as a starting point for the proposed transition because they are abundant in features and have a broad bandwidth. The proposed transition launches with 7 equal-length waveguide sections, as shown in Fig. 10b. Double ridges with two-step line sections were precisely placed over the rectangular waveguide aperture, acting as an impedance transformer, as illustrated in Fig. 10c. The structure’s dimensions were carefully selected to accommodate the TPGW line while maintaining proper alignment and impedance bandwidth.

A deep matching level of more than − 20 dB is required to achieve a relative bandwidth at 60 GHz. In practical terms, a back-to-back model is constructed to evaluate the performance of the proposed power divider structure with a conventional WR-15 waveguide, as shown in Fig. 9a, b. The performance of the proposed power divider is validated by fabricating a prototype. To measure the S-parameters, a Double Short-Short-Load-Through (SSLT) calibration was performed using an Anritsu 37397C Vector Network Analyzer (VNA) and two full-band WR15 right-angle adapters with a return loss (RL) of 20 dB, as shown in Fig. 9c, d. The calibration setup included two short circuits with offsets of 0 mm and 1.5 mm, as well as a precision termination with an RL of 30 dB. Measurements were conducted over the frequency range of 50–65 GHz. The simulation and measurement results demonstrate minor differences, which can be attributed to fabrication and assembly processes. In this case, surface roughness, which comprises microstructure and via holes that prevent current flow along ridge lines, causes differences between simulation and measured responses. Furthermore, the insertion loss distribution results show that the manufactured model’s 90-degree bends may exert an impact on performance. However, the simulation and measurement results are in good agreement, as demonstrated in Fig. 9e.

A 3-D view of the proposed 2$\times$2 ME antenna array fed by the designed differential power divider (DPD) is illustrated in Fig. 10a. The operation of individual elements is analyzed using the CST simulator with both time and frequency solvers, as well as HFSS, and certain components are validated through measurements. The next step involves testing the entire structure and verifying its overall performance. By combining the scattering matrices of the individual components with their simulated responses, the overall scattering parameters are obtained^43^. It is important to note that incorporating all the individual elements results in spikes in the structure’s scattering parameters, as shown in Fig. 10d. These spikes lead to performance degradation, making the structure unsuitable for practical implementation. While the combined structure reveals resonance spikes, the simulation results of the individual components do not exhibit any such resonant behavior. In practice, the simulations of the individual components do not face issues related to possible inconsistencies in the connections between components. Clearly, distinct reasons have emerged for the differences observed between the simulated results of the combined structure and those of the individual components. As a result, mathematical analyses are conducted to explore possible explanations for these discrepancies. A separate upcoming section will be dedicated to this comprehensive analysis.

## Mathematical analysis of cavity mode resonances excitation

**Fig. 11:**
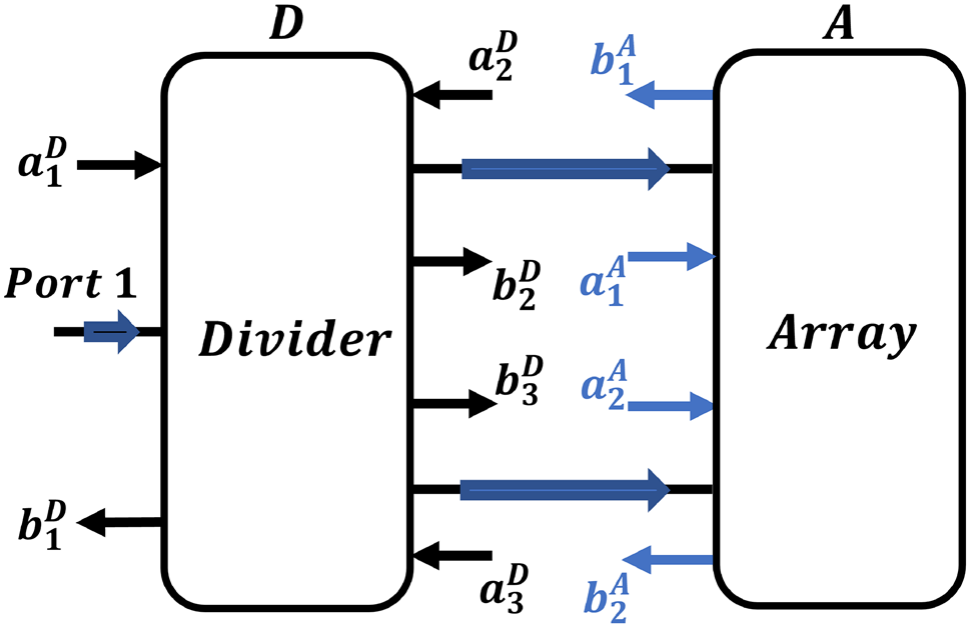
Schematic of the proposed antenna array structure.

The purpose of this section is to provide a mathematical analysis of how feasible mismatches among components impact the overall structure’s performance. It is possible to deduce the scattering parameter of the structure from the scattering parameters of its individual components as shown in Fig. 11.

To accommodate potential mismatches within the analysis, appropriate scattering parameter input variables are implied. As a way of simplifying the analysis, the following assumptions are made when writing the equations for the investigated schematic. This will be divided into:

1. *Differential power divider*
  - Perfect matching is observed at the input port of the divider, which can be depicted as follows:
    1 $$\begin{aligned} S_{11}^{D} = 0 \end{aligned}$$
  - The divider achieves an equal power division with a stable 180-degrees phase shift. As an outcome, the divider’s scattering matrix takes the pattern:
    2 $$\begin{aligned} S^{D} = \begin{bmatrix} 0 & \frac{1}{\sqrt{2}} & -\frac{1}{\sqrt{2}}\\ \frac{1}{\sqrt{2}} & \frac{1}{2} & -\frac{1}{2}\\ -\frac{1}{\sqrt{2}} & -\frac{1}{2} & \frac{1}{2} \end{bmatrix} \end{aligned}$$
2. *The 2 x 2 antenna array*
  1. The array with perfect matching and isolation (transmission parameters with the same magnitude)
  2. Variable $\alpha$ between the corresponding transmission channels expresses the array’s feasible discontinuity. The array’s transmission parameters are then attributed by:
    3a $$\begin{aligned} S_{21}^{A} = {\alpha _1} \end{aligned}$$
    3b $$\begin{aligned} S_{12}^{A} = {\alpha _2} \end{aligned}$$
    3c $$\begin{aligned} {\alpha _1}={\alpha _2}=\varvec{\alpha } \end{aligned},$$
    where (for reciprocity)
    4 $$\begin{aligned} S_{21}^{A} = S_{12}^{A} \end{aligned}$$

Using these hypotheses for all components’ simulated scattering matrices, the scattering parameters for Port 1 of the structure are as follows:

5 $$\begin{aligned} S_{11} = \frac{[S_{21}^{D} S_{11}^{A} + S_{31}^{D} S_{21}^{A} \;\;\;\;\;\; S_{21}^{D} S_{21}^{A} + S_{31}^{D} S_{11}^{A}] \begin{bmatrix} S_{21}^D\\[6pt] S_{31}^D\\ \end{bmatrix}}{[U] - [M][M']} \end{aligned}$$

The detailed derivation can be reviewed in the Appendix. Equation (5) can be used to calculate $S_{11}$ of the overall structure. The possibility of acquiring spikes of the magnitude of $S_{21}$ will be investigated. This will be examined by adjusting the variable $\alpha.$

**Fig. 12:**
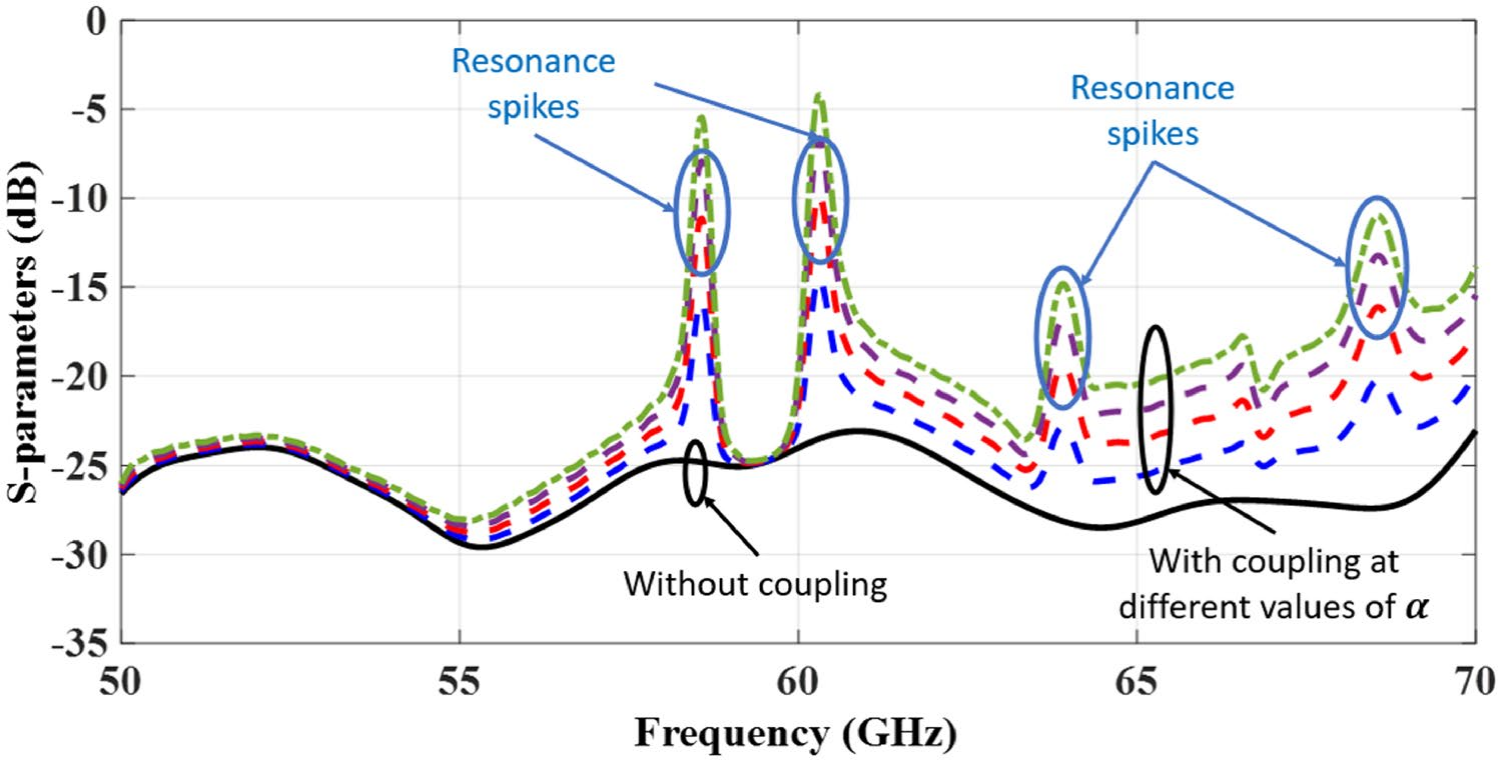
Illustration of the spikes condition with different values of $\alpha$.

A momentary and drastic degradation in $S_{21}$ coincides with a spike in the frequency response. Therefore, the variable $\alpha$ must be zero for $S_{21}$ to fade away. Aiming to eliminate the observed mutual coupling of relative locations and the interaction of sub-arrays, both of which have an impact on the overall response. To assess the spikes condition, the simulated responses of individual components are plugged into Eq. (5) to assess the spikes condition and the $S_{11}$ of the whole structure is depicted in Fig. 12. A preliminary investigation can provide a detailed explanation of the spikes situation. The entire structure port’s input signal is divided and forwarded to two two-element sub-arrays. Directed signals will have a coupling challenge since the 2 $\times$ 2 antenna array is not identical. Due to the coupling imbalances, the 2 $\times$ 2 antenna array does not work as expected, which influences the entire structure’s response. In fact, the transmission coefficient is a frequency-dependent variable; hence, the resonance condition occurs at particular frequencies, as observed by the spikes at these frequencies. It ought to be emphasised that the illustrated spikes within this analysis differ slightly from the simulated results of the previous section. It is due to the inclusion of ideal assumptions for a 2 $\times$ 2 antenna array. A full analysis would take into account the coupling issue caused by the antenna array’s mismatches, resulting in a more general resonance condition that could be obtained. Therefore, the main objective is to properly assess the concepts rather than complete a full analysis of the structure difficulties.

**Fig. 13:**
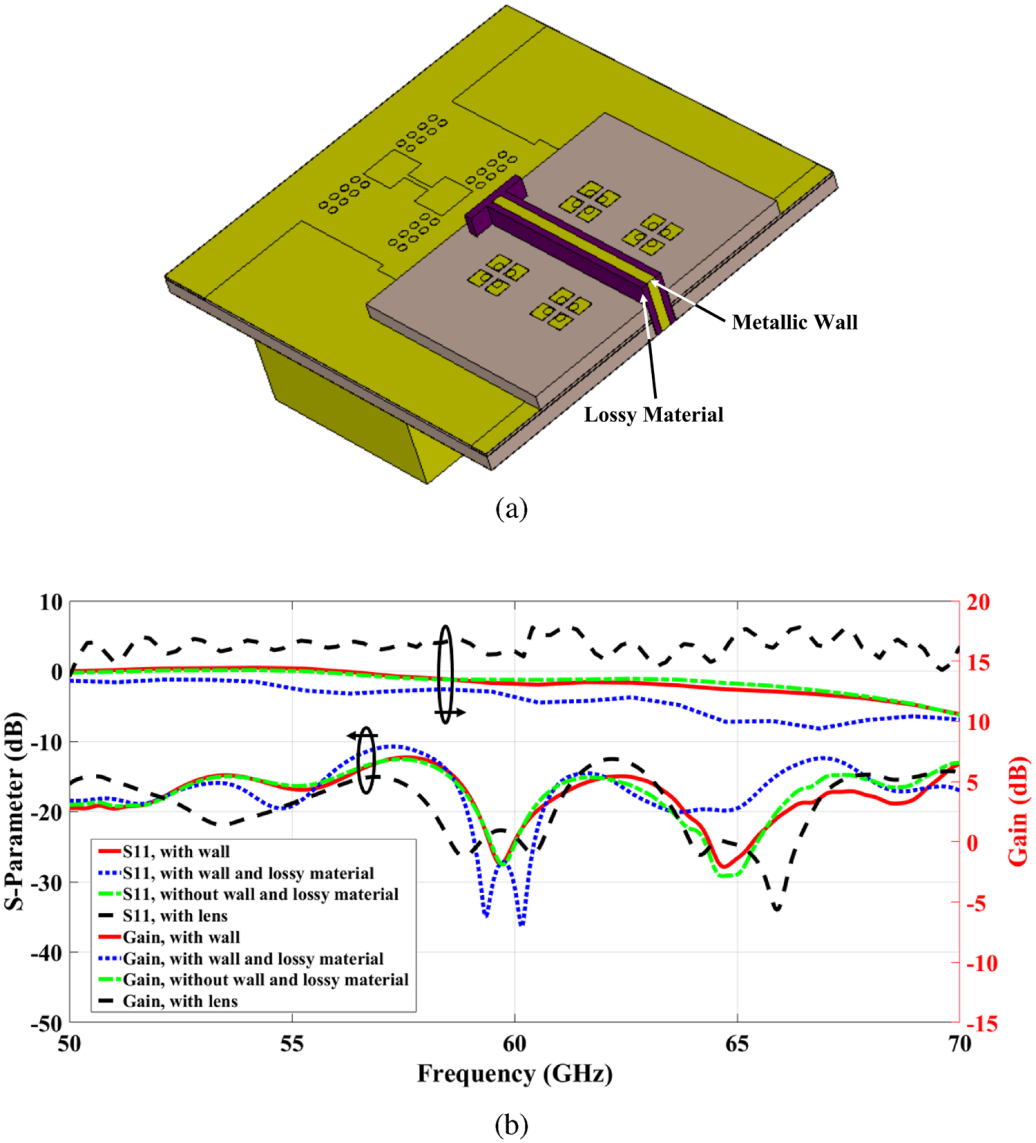
2 $\times$ 2 ME dipole antenna array structure. (**a**) 3-D view of the whole structure with metallic wall and lossy material. (**b**) Simulation results and comparison. For clarity, the lens layer’s ground has been hidden.

To transcend the resonance condition, the coupling issue must be resolved in order to avoid the signal from intersecting and resonating. The following section aims to break the virtual cavity and eliminate spikes by separating the two two-element antenna arrays.

## Modified array structure: mutual coupling solution

Minimizing mutual coupling between antenna elements in 60-GHz antenna arrays is essential due to its significant impact on port impedance and overall performance. Corrugated structures have been employed to reduce mutual coupling between two antenna elements^44^. Isolation enhancement via a corrugated structure of a specific height shows a noticeable effect on the operating frequency. Corrugated walls can be modeled as a parallel-plate transmission line shorted at the bottom. Thus, the bottom short-ended circuit transforms into a capacitive surface, owing to the corrugation height, which suppresses TM surface waves^45^. Additionally, a simple and cost-effective solution for minimizing coupling between two slot antennas operating at 60 GHz is proposed^46^. This approach limits coupling by removing a portion of the TPGW’s upper metallic plate between the two slots. In light of these methods, we aim to control the coupling issue in our proposed antenna array modification.

**Fig. 14:**
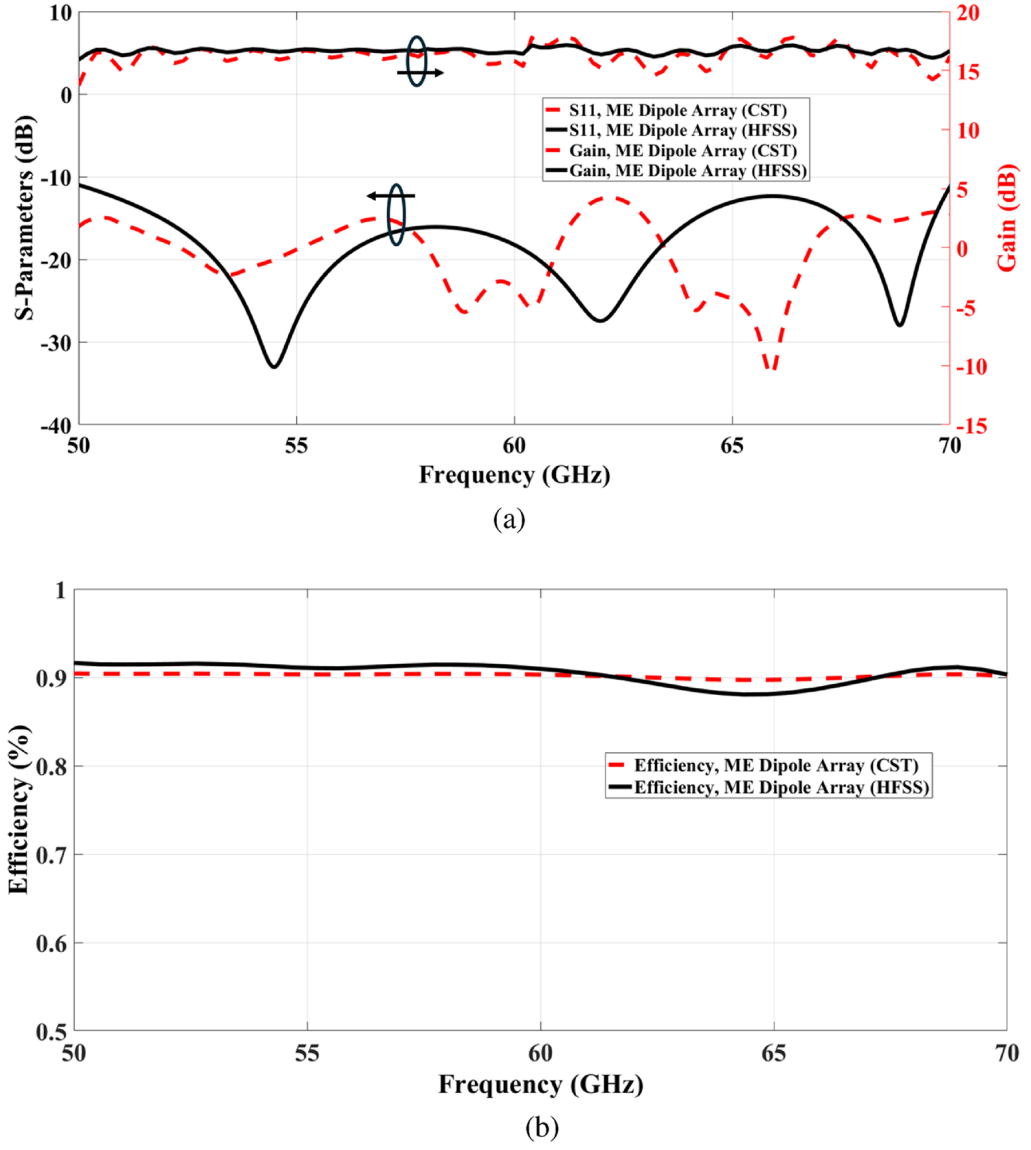
2 $\times$ 2 ME dipole antenna array simulation results and validation. (**a**) Reflection coefficient and gain. (**b**) Efficiency.

**Fig. 15:**
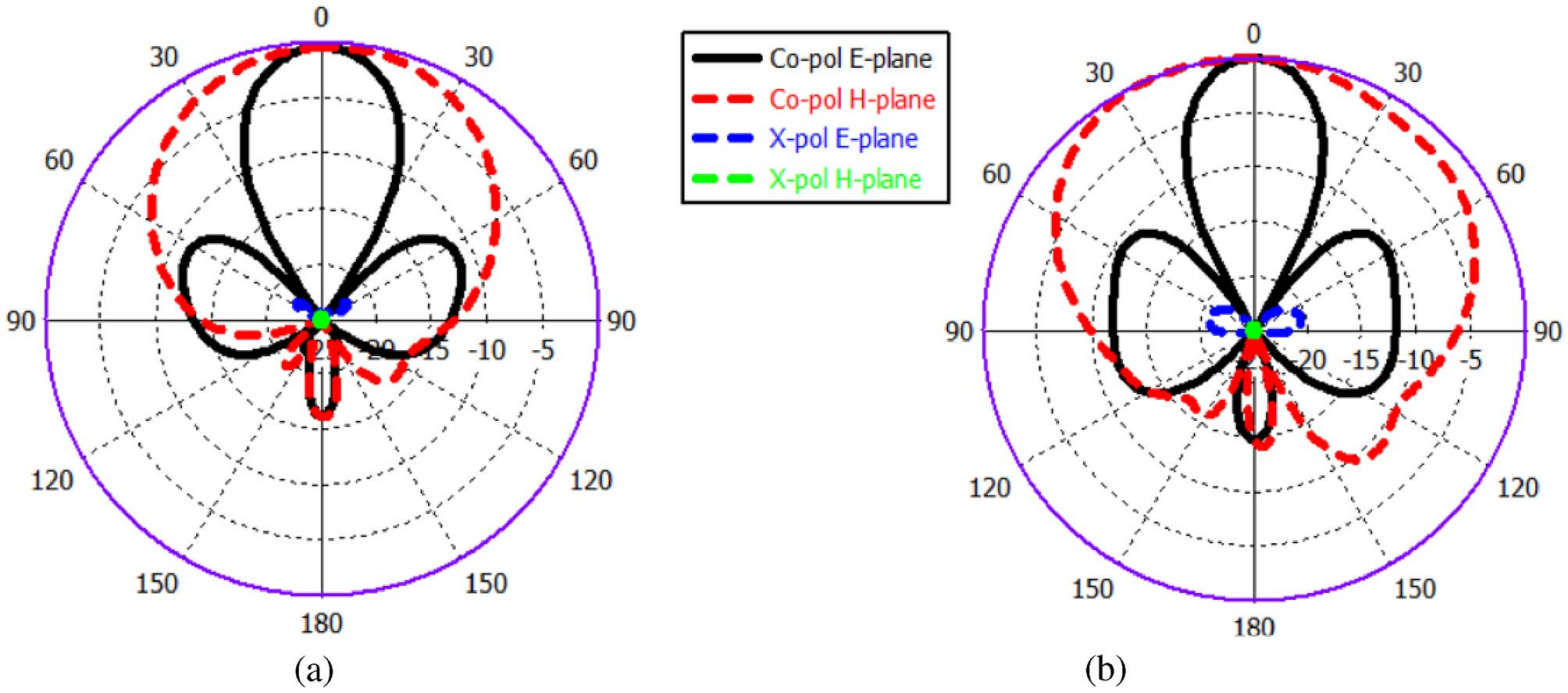
Radiation patterns of the 2x2 ME dipole antenna array at 60 GHz. (**a**) CST. (**b**) HFSS.

The details of the design for a 2$\times$2 antenna array with small separation and minimal mutual coupling are presented in Fig. 13a. Without the virtual cavity, the preceding resonance condition cannot be maintained, which prevents signal bouncing. Mutual coupling primarily arises due to the common TPGW top metallic plate and waves travelling between two nearby slots^47^. Additionally, coupling caused by surface waves in the substrate is not negligible for the physical substrate of the two-element array. A simple and efficient approach involves removing a vertical portion of the entire layer to separate the two-element sub-arrays. This effectively reduces surface currents in the TPGW top metallic plate between the nearby elements, as well as surface waves within the substrate. Moreover, a wall of a certain height with a surrounding layer of lossy material is added to ensure no coupling between the sub-arrays occurs. Figure 13b shows the return loss of the modified antenna array, where no spikes are detected.

The proposed antenna’s performance has been developed and assessed using (CST) Microwave Studio, a full-wave EM simulator. The simulated result accomplished through the High Frequency Structural Simulator Ansoft (HFSS) software is also provided for comparison and validation. The simulated reflection coefficient |S11| and the gain of the proposed antenna array via CST and HFSS is shown in Fig. 14a. The results reveal that simulated return loss bandwidth (|S11| < − 10 dB) operates from 50 GHz to 70 GHz, covering the 60 GHz frequency spectrum. In addition to a superior performance in terms of bandwidth and gain, the proposed design can demonstrate superior radiation characteristics, with a large radiation efficiency of more than 90% as depicted in Fig. 14b, appropriate for mm-Wave applications. Furthermore, The simulated far-field radiation pattern of the antenna at 60 GHz illustrated in Fig. 15. The figure demonstrates that the antenna is characterised by a narrower beam in the yz plane (E-plane), whereas a wider beam is produced in the xz plane (H-plane).

The S-parameter, gain, efficiency, and radiation pattern have been validated utilising the CST and HFSS simulators. At high frequencies, there are a few variations in the simulated results between the CST and HFSS simulators. Meanwhile, there appears to be satisfactory agreement between CST and HFSS data responses, with an acceptable inequality, which could be attributed to differences in simulator conditions.

## Evaluation and comparison

The performance of the proposed ME dipole antenna array is evaluated by comparing it with other recently developed antennas and arrays utilizing different technologies. The comparison explores several potentials of the presented antenna array structure, commencing with an evaluation of the TPGW-based ME antenna array’s capabilities. Table 5 summarises the comparison of fundamental characteristics and performance with reported designs in the mm-wave spectrum, the majority of which are at 60 GHz. This can be demonstrated by providing an alternating series of array structures and their performance improvements over the past decade. It is worth mentioning that the most reported designs for the comparison present plus 4-element or 4$\times$4 antenna arrays or more. This emphasises the significance of the design provided and the extent to which it presents superior performance beyond the related work, particularly in the 60-GHz frequency band. In comparison to the 4$\times$4 patch antenna array based on LTCC technology in^49^, which provides high gain for the 60 GHz band; however, these designs often have significant production expenses and complex assembly operations. Moreover, the demonstrated array features a larger bandwidth, a lower profile, better radiation efficiency, and a greater gain. Compared with the 2-slot antenna arrays using SIW in^50^, the presented array provides a lower profile and a greater gain. The provided array outperforms another SIW-based cavity-backed 4$\times$4 patch array in^51^ in terms of impedance bandwidth, profile, and radiation efficiency. While SIW-based structures have high power handling capabilities, leakage losses pose a challenge due to the separation of via holes located on the top planes. Furthermore, due to the presence of dielectric in the waveguide design, dielectric losses will occur; the dominant mode is a TE mode, which has more dispersion. In addition, SIW feeding networks have a large insertion loss which exceeds 1 dB due to dielectric losses, resulting in additional losses. Moreover, the bandwidth is considerably wide at the expense of a big size and the complex, expensive fabrication process. As a result, it can be difficult to incorporate with the monolithic microwave integrated circuit. On the other hand, the TPGW guiding structure has the advantage at mm-wave frequencies, which has been proposed to relatively reduce overall loss and allow for signal transmission with minimal dispersion. Moreover, the proposed structure is printed on a substrate which can be integrated with other planner circuits.

In^53^, a multilayered LTCC was employed to design a slot patch antenna array backed by SIW cavity. This approach combines extensive manufacturing costs and dielectric losses. MMoreover, the suggested antenna has a clear advantage in accordance with bandwidth, efficiency, and gain. Furthermore, the SIW-based 2$\times$2 aperture coupled ME antenna array that was implemented in^52,54^ is easily integrated with differential mm-wave devices and has adequate impedance and bandwidth to guarantee the requirements for 60 GHz communication. Since more losses are presumed to be experienced when SIW technologies as a result of the dielectric material being used in contrast with gap waveguide technology.

On the other hand, a broad 3-dB gain bandwidth is achieved in^55^ by combining the Fabry-Perot cavity (FPC) above a slot antenna operating at 60 GHz and fed by PRGW. It can be observed that the proposed ME dipole antenna array has a wider impedance bandwidth, higher gain, a more compact profile, and better radiation efficiency. Besides, the proposed structure provides a ME dipole antenna based on TPGW technology that has stable radiation characteristics over a wide bandwidth. Furthermore, 30-GHz 1$\times$4 ME dipole antenna arrays excited by one slot or two slots coupled to a fork-shaped PRGW are exhibited in^56,57^. Moreover, antenna gain is enhanced by incorporating vertically three layers of split ring resonators (SRR) in front of the ME dipole. The aforementioned techniques feature bulky structures with large sizes that comprise of 7 layers. Therefore, the provided antenna array has a step over these structures with a lower profile in terms of structure layers, greater gain, and radiation efficiency, as well as a relatively broad bandwidth. A large 16$\times$16 ME-dipole antenna array fed by microstrip ridge gap waveguide (MRGW) through a narrow slot has been introduced in^58^. In the described example, the array elements are electrically fed by the MRGW, affording more flexibility for developing complex feeding networks. However, MRGW fails to provide better radiation efficiency due to the presence of dielectric material, and its design for complicated feeding networks is quite challenging, perhaps requiring comprehensive full-wave design and optimisation.

A wideband 2$\times$4-slot array antenna based on GW technology is demonstrated^59^. GW technology is employed to prevent EM leakage between metallic blocks. In the mentioned design, a further cavity power divider layer is added between the radiation slots and the power divider’s cavity layers to regulate the amplitude and phase distributions in the desired wideband region. This can eliminate the array’s grating lobes. Despite the high performance of the array, this approach sacrifices antenna array efficiency. Moreover, our proposed array outperforms the mentioned structure in terms of impedance bandwidth and the gain as well. Another GW-based antenna array, a four-element wideband mm-Wave array based on the PRGW, is presented^60^. That indicates how this technique has already been utilised to develop feeding networks for antenna arrays. Furthermore, the PRGW is considerably lighter and easier to fabricate and integrate, leading it to be a more desirable choice. When compared to the several demonstrated approaches, this model failed to provide the expected additional performance.

The potential of GW technology to develop and operate wideband low antenna arrays with high gain and wide bandwidth which fulfill the necessary requirements of mm-Wave communications. Since the feeding network is the reason for the majority of loss in an array antenna, RGWs are used to avoid the feeding losses, resulting in a high-performance antenna array. The design a high-gain cavity-backed slot array with low-loss RGW-based feeding network is presented^61^. Although the large array antenna elements are owing to high gain, the bandwidth of the antenna of the array and its efficiency are considerably restricted.

**Table 5:** Comparison of the antenna gain and operating band for different types of antenna technologies.

Ref. (year)	Frequency (GHz)	Technology	Antenna type	Impedance bandwidth	Gain (dBi)	Max. radiation efficiency	Area (mm⌃2)	No. of layers
^49^(2013)	60	Multilayer LTCC	4 x 4 L-probe Patch Antenna Array	29%	17.5	85%	2.8 $\lambda _{0}$ x 2.8 $\lambda _{0}$	2
^50^(2018)	60	SIW	4- Slot Antenna Array	35%	$\sim$12.5	92%	3.6 $\lambda _{0}$ x 3.4 $\lambda _{0}$	2
^51^(2014)	60	SIW	4 x 4 cavity-backed Patch Antenna Array	22.6%	$\sim$19.6	80%	3.26 $\lambda _{0}$ x 3.42 $\lambda _{0}$	4
^52^(2015)	60	SIW	2 x 2 Aperture coupled ME Dipole Antenna Array	22%	12.5	70%	1.4 $\lambda _{0}$ x 1.4 $\lambda _{0}$	3
^53^(2014)	60	LTCC/SIW	2 x 2 Dual Resonant Slot-Patch Antenna Array	23%	9	NA	1.94 $\lambda _{0}$ x 1.3 $\lambda _{0}$	2
^54^(2017)	60	SIW	2 x 2 Aperture coupled ME Dipole Antenna Array	28.7%	14	$\sim$90%	1.4 $\lambda _{0}$ x 1.4 $\lambda _{0}$	3
^55^(2017)	60	PRGW	Slot Antenna Loaded with 2 layers PRS	20.4%	15.6	89%	1.66 $\lambda _{0}$ x 1.4 $\lambda _{0}$	3
^56^(2016)	30	PRGW	1 x 4 ME Dipole with Meta Lens Loading	35.2%	17.5	85%	2.4 $\lambda _{0}$ x 3.8 $\lambda _{0}$	7
^57^(2020)	30	PRGW	DCS ME Dipole Loaded with 3 layers SRR	50%	14.2	90%	1.1 $\lambda _{0}$ x 1 $\lambda _{0}$	7
^58^(2020)	60	MRGW	16 x 16 ME Dipole Antenna Array	19%	30	70%	1.52 $\lambda _{0}$ x 1.52 $\lambda _{0}$	3
^59^(2021)	30	GW	2 x 4 Slot Sub- Array Antenna	26.4%	16.7	76.3%	–	4
^60^(2023)	30	PRGW	1 $\times$ 4 Shorted Circular Patch Array Antenna	10.3%	12.6	–	1.9 $\lambda _{0}$ x 3.3 $\lambda _{0}$	5
^61^(2024)	30	RGW	16 $\times$ 16 Slot Antenna Array	17%	28.9	65%	11.5 $\lambda _{0}$ x 11.5 $\lambda _{0}$	3
This work	60	TPGW	2 x 2 ME Dipole Antenna Array with Lens Loading	> 33.3%	> 18	90%	1.6 $\lambda _{0}$ x 1.4 $\lambda _{0}$	4

As a result, the proposed array is designed based on different approaches, where the ME dipoles are proximity fed by a TPGW feeding network through a thin dielectric-filled gap using trapping GW. Moreover, the antenna is integrated with a perforated dielectric substrate layer lens to improve the gain. The utilization of TPGW technology in this study offers several advantages at mm-wave frequencies, primarily due to the minimal loss attributed to the wave traveling within the air gap instead of the dielectric, resulting in higher radiation efficiency compared to other technologies. it is important to highlight that our design has a lower number of array elements than the reported structures, while it provides significant performance in terms of impedance bandwidth, gain, efficiency, and smaller size. According to the authors’ best knowledge, a high-gain, wideband ME dipole antenna array based on TPGW at the 60-GHz mm-Wave spectrum has been developed that is not detectable in the previously discussed approaches. In addition, a thorough mathematical analysis is performed for the excited gravity mode, which helps better understand the work.

As a summary, the proposed design is compact and cost-effective, while its use of TPGW technology ensures superior bandwidth and radiation efficiency. By minimizing dielectric loss, the air-filled gap in the TPGW enhances performance, making the proposed antenna highly efficient. The enhanced gain and wideband impedance matching, coupled with the compact size, make it a superior candidate compared to other designs at the same frequency range. These factors contribute to the potential use of this antenna in future mm-wave communication systems, where the combination of high gain, wide bandwidth, and minimal loss is critical. In comparison to other related antenna array designs, this study demonstrates a substantial improvement in bandwidth, efficiency, and gain, highlighting the significance of using TPGW technology. These results establish the proposed antenna as an exceptional solution for mm-wave applications, with performance metrics exceeding those reported in the literature.

## Conclusion

The ME dipole antenna fed by a bow-tie slot through a TPGW is implemented. The antenna is designed for broadband and high-gain mm-wave applications in the 60 GHz frequency spectrum. The proposed antenna presents a compromise between losses minimization and the structure simplicity. It has been demonstrated that applying the trapped PGW approach with filling partial air gaps in a traditional PGW can eliminate frustrating assembly operations for standalone high-frequency circuits. It covers a bandwidth with |S11| < – 10 dB of 33.3 % from 50 to 70 GHz with a maximum gain of 11 dBi over the operating bandwidth. This can be accomplished by using a perforated dielectric substrate layer lens to improve the antenna gain for operation over the whole operating bandwidth. The two-way broadband power divider is applied for the 2-element antenna sub-array. Employing the 2-element sub-array, larger array antennas for high-gain applications can be realised. The ME-dipole sub-arrays and out-of-phase power divider with WR-15 standard interface are designed and investigated individually. A methodical design process is used for obtaining initial design parameters. Simulations and measurements have been employed to assess the performance of individual components. Implementation of this method resulted in resonance spikes at several frequencies in the S-parameter profile. Mathematical investigation revealed that the resonance condition stems from disparities between practical components. A potential approach that involves dissolving the resonance loop to separate the two 2-element sub-arrays of the whole structure has been demonstrated. The 2$\times$2 array antenna has a deep level matching bandwidth of 33.3%, a peak gain greater than 18 dBi, and a simulated radiation efficiency of around 90% across the entire band. The presented antenna is appropriate for 5G applications as it satisfies market and technical expectations for broad bandwidths, stable high gain, symmetrical radiation patterns, minimal loss, compact dimensions, and easy implementation.

## Supplementary Information

Supplementary Information.

## Data Availability

All data generated or analysed during this study are included in this published article [and its information files].
