# Supplementary material for: Design and analysis of a 60 GHz high gain wideband magneto electric dipole antenna array based on trapped printed gap waveguide technology
Source: Sci Rep. 2025 Jul 2;15:23649. doi: 10.1038/s41598-025-08589-9 (PMC12222962; doi:10.1038/s41598-025-08589-9)
Supplement: Supplementary file 1 — Supplementary Information. [file 41598_2025_8589_MOESM1_ESM.pdf]

## Appendix

A scattering matrix is used to represent scattering parameters in N-port networks.

$$[b] = [S][a] \tag{A.1}$$

, where $a_i$ and $b_i$ denote the normalised incident and reflected wave amplitudes, respectively. The matrix's order is determined by the number of exterior ports: three for the dividers and two for the antenna array. Formula (A.1) extends to each individual component in the Fig. 11 schematic along with the entire structure.

The following set of simplified formulas result from utilising the first hypothesis of both the divider and the antenna array as stipulated in Section IV:

$$b_2^D = a_1^A \tag{A.2a}$$

$$a_2^D = b_1^A \tag{A.2b}$$

$$b_3^D = a_2^A \tag{A.2c}$$

$$a_3^D = b_2^A \tag{A.2d}$$

Accordingly, utilising Formula (**??**) as the first hypothesis of the power divider, the divider can be characterized as follows:

$$\begin{bmatrix} b_1 \\ b_2 \\ b_3 \end{bmatrix} = \begin{bmatrix} 0 & S_{12} & S_{13} \\ S_{21} & S_{22} & S_{23} \\ S_{31} & S_{32} & S_{33} \end{bmatrix} \begin{bmatrix} a_1 \\ a_2 \\ a_3 \end{bmatrix} \tag{A.3}$$

Reflections would not occur at the interconnect points due to the identical dimensions of the ports of the connected components. As a consequence, the signals at such points are the same. In this case, (A.3) can be written in terms of array signals. And the formula for the divider $D$ seems to be:

$$\begin{bmatrix} b_1^D \\ b_2^D \\ b_3^D \end{bmatrix} = \begin{bmatrix} 0 & S_{12}^D & S_{13}^D \\ S_{21}^D & S_{22}^D & S_{23}^D \\ S_{31}^D & S_{32}^D & S_{33}^D \end{bmatrix} \begin{bmatrix} a_1^D \\ b_1^A \\ b_2^A \end{bmatrix} \tag{A.4}$$

and for the array $A$ seems to be:

$$\begin{bmatrix} b_1^A \\ b_2^A \end{bmatrix} = \begin{bmatrix} S_{11}^A & S_{12}^A \\ S_{21}^A & S_{22}^A \end{bmatrix} \begin{bmatrix} a_1^A \\ a_2^A \end{bmatrix} \tag{A.5}$$

, and for reciprocity seems to be, for divider $D$:

$$\begin{bmatrix} b_1^D \\ b_2^D \\ b_3^D \end{bmatrix} = \begin{bmatrix} 0 & S_{21}^D & S_{31}^D \\ S_{21}^D & S_{22}^D & S_{32}^D \\ S_{31}^D & S_{32}^D & S_{33}^D \end{bmatrix} \begin{bmatrix} a_1^D \\ b_1^A \\ b_2^A \end{bmatrix} \tag{A.6}$$

, and for array $A$:

$$\begin{bmatrix} b_1^A \\ b_2^A \end{bmatrix} = \begin{bmatrix} S_{11}^A & S_{21}^A \\ S_{21}^A & S_{22}^A \end{bmatrix} \begin{bmatrix} a_1^A \\ a_2^A \end{bmatrix} \tag{A.7}$$

, and for ideal case of symmetry seems to be, , for divider $D$:

$$\begin{bmatrix} b_1^D \\ b_2^D \\ b_3^D \end{bmatrix} = \begin{bmatrix} 0 & S_{21}^D & S_{31}^D \\ S_{21}^D & S_{22}^D & S_{32}^D \\ S_{31}^D & S_{32}^D & S_{22}^D \end{bmatrix} \begin{bmatrix} a_1^D \\ b_1^A \\ b_2^A \end{bmatrix} \tag{A.8}$$

, and for array $A$:

$$\begin{bmatrix} b_1^A \\ b_2^A \end{bmatrix} = \begin{bmatrix} S_{11}^A & S_{21}^A \\ S_{21}^A & S_{22}^A \end{bmatrix} \begin{bmatrix} a_1^A \\ a_2^A \end{bmatrix} \tag{A.9}$$

Substituting for the array signals $b_1^A$ and $b_2^A$ from (A.2b) and (A.2d) produces the following equation for the divider $D$:

$$\begin{bmatrix} b_1^D \\ b_2^D \\ b_3^D \end{bmatrix} = \begin{bmatrix} 0 & S_{21}^D S_{11}^A + S_{31}^D S_{21}^A & S_{21}^D S_{21}^A + S_{31}^D S_{11}^A \\ S_{21}^D & S_{22}^D S_{11}^A + S_{32}^D S_{21}^A & S_{22}^D S_{21}^A + S_{32}^D S_{11}^A \\ S_{31}^D & S_{32}^D S_{11}^A + S_{22}^D S_{21}^A & S_{32}^D S_{21}^A + S_{22}^D S_{11}^A \end{bmatrix} \begin{bmatrix} a_1^D \\ a_1^A \\ a_2^A \end{bmatrix} \tag{A.10}$$

To facilitate manipulation, (A.10) will be divided into two segments. Therefore, First line of (A.10) can be expressed as:

$$b_1^D = \begin{bmatrix} S_{21}^D S_{11}^A + S_{31}^D S_{21}^A & S_{21}^D S_{21}^A + S_{31}^D S_{11}^A \end{bmatrix} \begin{bmatrix} a_1^A \\ a_2^A \end{bmatrix} \tag{A.11}$$

which provides the structure's output wave at Port 1. The remaining two lines of (A.10) can be merged as follows:

$$\begin{bmatrix} a_1^A \\ a_2^A \end{bmatrix} = \begin{bmatrix} S_{21}^D \\ S_{31}^D \end{bmatrix} a_1^D + \begin{bmatrix} S_{22}^D & S_{23}^D \\ S_{23}^D & S_{22}^D \end{bmatrix} \begin{bmatrix} S_{11}^A & S_{21}^A \\ S_{21}^A & S_{11}^A \end{bmatrix} \begin{bmatrix} a_1^A \\ a_2^A \end{bmatrix} \tag{A.12}$$

And can be rewritten as:

$$\begin{bmatrix} a_1^A \\ a_2^A \end{bmatrix} = \begin{bmatrix} S_{21}^D \\ S_{31}^D \end{bmatrix} a_1^D + [M][M'] \begin{bmatrix} a_1^A \\ a_2^A \end{bmatrix} \tag{A.13}$$

, where

$$[M] = \begin{bmatrix} S_{22}^D & S_{23}^D \\ S_{23}^D & S_{22}^D \end{bmatrix} \tag{A.14a}$$

$$[M'] = \begin{bmatrix} S_{11}^A & S_{21}^A \\ S_{21}^A & S_{11}^A \end{bmatrix} \tag{A.14b}$$

Formula (A.12) can be resolved for the vectors $[a_1^A \; a_2^A]^T$ in terms of the structure input wave $a_1^D$ as follows:

$$\begin{bmatrix} a_1^A \\ a_2^A \end{bmatrix} = \begin{bmatrix} S_{21}^D \\ S_{31}^D \end{bmatrix} a_1^D \; x \; ([U] - [M][M'])^{-1} \tag{A.15}$$

, where [U] is the unity matrix. Substituting for these vectors in (A.11) gives the following scattering parameter of the whole structure:

$$b_1^D = \frac{\begin{bmatrix} S_{21}^D S_{11}^A + S_{31}^D S_{21}^A & S_{21}^D S_{21}^A + S_{31}^D S_{11}^A \end{bmatrix} \begin{bmatrix} S_{21}^D \\ S_{31}^D \end{bmatrix} a_1^D}{([U] - [M][M'])} \tag{A.16}$$

The scattering parameters of the structure should indeed be retrieved to determine the impact of components imbalance. For Port 1, the $S_{11}$ are:

$$S_{11} = \frac{b_1^D}{a_1^D} = \frac{\begin{bmatrix} S_{21}^D S_{11}^A + S_{31}^D S_{21}^A & S_{21}^D S_{21}^A + S_{31}^D S_{11}^A \end{bmatrix} \begin{bmatrix} S_{21}^D \\ S_{31}^D \end{bmatrix}}{([U] - [M][M'])} \tag{A.17}$$
